# Mental Health and Recreation Opportunities

**DOI:** 10.3390/ijerph17249338

**Published:** 2020-12-14

**Authors:** Kyung Hee Lee

**Affiliations:** Department of Recreation, Parks and Leisure Services Administration, Central Michigan University, Mount Pleasant, MI 48859, USA; lee8k@cmich.edu

**Keywords:** mental health days, recreation opportunities, opportunity theory, geographically weighted regression, walkability

## Abstract

The environment has direct and indirect effects on mental health. Previous studies acknowledge that the poor design of communities and social environments leads to increased psychological distress, but methodological issues make it difficult to draw clear conclusions. Recent public health, leisure and recreation studies have tried to determine the relationship between recreation opportunities and mental health. However, previous studies have heavily focused on individual contexts rather than national or regional levels; this is a major limitation. It is difficult to reflect the characteristics of community environments effectively with such limited studies, because social environments and infrastructure should be analyzed using a spatial perspective that goes beyond an individual’s behavioral patterns. Other limitations include lack of socioeconomic context and appropriate data to represent the characteristics of a local community and its environment. To date, very few studies have tested the spatial relationships between mental health and recreation opportunities on a national level, while controlling for a variety of competing explanations (e.g., the social determinants of mental health). To address these gaps, this study used multi-level spatial data combined with various sources to: (1) identify variables that contribute to spatial disparities of mental health; (2) examine how selected variables influence spatial mental health disparities using a generalized linear model (GLM); (3) specify the spatial variation of the relationships between recreation opportunities and mental health in the continental U.S. using geographically weighted regression (GWR). The findings suggest that multiple factors associated with poor mental health days, particularly walkable access to local parks, showed the strongest explanatory power in both the GLM and GWR models. In addition, negative relationships were found with educational attainment, racial/ethnic dynamics, and lower levels of urbanization, while positive relationships were found with poverty rate and unemployment in the GLM. Finally, the GWR model detected differences in the strength and direction of associations for 3109 counties. These results may address the gaps in previous studies that focused on individual-level scales and did not include a spatial context.

## 1. Introduction

Recent leisure studies have tried to determine the relationship between mental health and access to recreation opportunities [1,2,3,4,5]. Most researchers have shown an increased interest in the relationship between mental health and recreational service provision at specific locations rather than regional systems.

Opportunity theory implies that “participation in different forms of recreation depends on their availability” [6]. A considerable number of studies have identified that individuals with physical and financial access to recreation resources can experience a higher quality of life through participation in recreation activities from a long-term perspective [7,8,9,10,11,12]. The previous leisure studies using opportunity theory have identified environmental injustices associated with unequal access to parks and inadequate recreation opportunities for underserved populations that affect the physical health of the community at large [13,14,15]. Specifically, low-income and minority populations are most dependent on having access to free, accessible, and quality open spaces for recreation. As is cited previous studies, the “physical and psychological benefits of using parks and greenways are particularly prominent” [16,17] and “visiting parks and connecting with nature are associated with improved mental health” [18,19]. Further benefits include reduced stress [20,21], optimistic moods and psychological well-being [22,23], enjoyment of landscape beauty [24] and place attachment [25].

In addition, infrastructure does more than protect people from natural disaster; it can have a positive impact on public health [26]. The infrastructure of streets, neighborhoods, and metropolitan areas is widely described in previous studies, but the positive role of park walkability is less discussed [27,28]. There are a wide range of recreational open spaces, and though the national, state, and regional open spaces are important, the role of local recreational infrastructure should not be overlooked. Specifically, previous studies found that neighborhood environments can influence the levels of walking for recreation; however, most of these studies were limited to low-density urban areas [29,30,31]. Recreation opportunities have an important link to the proximity of recreation facilities. Proximity is principally measured by two key land-use factors: density and land use mix. The more compact definition of proximity is short distances between destinations. “Distances of less than a half mile between residences”, stores, stations and bus stops are desirable for walking [32,33,34]. Historically, guidelines in the United States and Canada defined a range of 300–900 m (0.19–0.56 miles) as a “walkable distance” [35]. Floyd and his colleagues insisted that most users of parks come from a very localized area, usually less than a quarter-mile [36]. Critics have also argued that surveys provide an inaccurate measure of proximity; this methodological limitation could be reduced using spatial data. Methodologically, only a few studies have examined park walkability and mental health at macro-level scales, such as across counties or the national level [37,38]. Many of these studies exploring recreation participation have focused on individual-level research and were conducted on a local scale [39], thus showing limited support for the theory at a regional or national level. Furthermore, there are differences in the association between recreation opportunities and socioeconomic status (SES). Therefore, it is important to test relationships in combination with socioeconomic status. This is because one major drawback of public health studies in the U.S. is that “there has been very limited public health effort that focuses on improving SES in general and reducing SES disparities in particular” [40].

Until now, there have been no specific evidence-based studies to support recreation opportunity theory in relation to mental health using a spatial multi-level analysis. The main purposes of this study are the following: (1) detecting spatial clustered patterns of mental health at a national level; (2) determining how recreation opportunities (park walkability) contribute to mental health at a national level after controlling for socioeconomic variables; (3) analyzing the spatial variation of relationships between recreation opportunities (park walkability) and mental health at the county level in the continental United States.

To narrow the gaps in previous research, this study used spatial analysis and macro-level data to test the relationship between mental health and walkable park availability with the socioeconomic information of communities to indicate mental health disparities across the country. Through the lens of opportunity theory, the models developed in this study were tested at the county level to evaluate the role of recreation opportunities (accessibility and density) and competing factors (socioeconomic and environmental) on the condition of mental health.

## 2. Method

Social science studies commonly use global statistics to detect social trends through aggregated data. However, global statistics have limitations when used in data analysis on a regional scale. Various scale-related problems have been identified in the analysis of spatially aggregated data [41]. First, Simpson’s Paradox emphasizes the risk of analyzing aggregate data “where the aggregation is over population subgroups; the paradox applies equally to spatial data where the aggregation is over locations” [42]. Second, the modifiable areal unit problem (MAUP) is a problem that is isomorphous to the statistical inference problem. The MAUP “originates from the fact that areal units are usually randomly determined and modifiable, in the sense that they can be aggregated to form units of different sizes or spatial arrangements” [43]. The MAUP has two linked but idiosyncratic components: the scale and the zoning problems [44]. Openshaw et al. explained the scale problem as “the variation in results that may be obtained when the same areal data are combined into sets of increasingly larger areal units of analysis” and the zoning problem as “variations in results due to alternative units of analysis where n, the number of units, is constant” [45]. Specifically, later studies outlined some potential solutions: “increasing the aggregation of units by increasing the area covered by the units decreases the variance in the data between the units” for the scale problem, and “rezoning the areas contained by each unit while holding the total number of units the same can impact both the mean and variance of any measured data” for the zoning problem [46].

It is crucial to reduce these problems by reflecting spatial variations. The benefit of employing a locally calibrated regression model is that it can be used to search for spatial disparities in regional trends. In this sense, geographically weighted regression (GWR) is effective in diminishing the effects of Simpson’s paradox and MAUP.

The study area was the continental U.S., including 48 states and 3109 counties. County boundary data were obtained from the Census Bureau website and converted into queen-contiguity-based weights, as well as higher order contiguity for cluster analysis and spatial effect estimation.

### 2.1. Dependent Variable

Research data was drawn mainly from the Behavioral Risk Factor Surveillance System (BRFSS). This study used a “health days measure” to represent the mental health condition. In the healthy days core module, there were four main questions related to mental and physical health conditions. Specifically, the dependent variable stemmed from the following question: “Now thinking about your mental health, which includes stress, depression, and problems with emotions, for how many days during the past 30 days was your mental health not good? [the number of days in the past 30]” [47]. Prior to 2014, BRFSS data focused on only state-level estimates. The CDC (Centers for Disease Control and Prevention) has started manipulating a multilevel modeling approach based on respondent answers, individual-level socio-demographic information, county-level economic status, and both county- and state-level contextual effects [48]. These changes may provide researchers with the most accurate estimates of community health conditions. This study used county-level mentally unhealthy days (age-adjusted) as the dependent variable. In addition, the mean score of poor mental health was calculated for each county as reported in the past 30 days.

### 2.2. Independent Variables

Based on previous studies and conceptual framework, explanatory variables were carefully selected for the study. Density of recreation and sports facilities and park walkability were used to represent county-level recreation opportunities. In terms of SES, various economic variables (poverty rate, unemployment, and family median income) and demographic variables (education attainment, age, and family structure) were adopted. The level of urbanization (percentage of rural area), percentage of lands for future development, density of sports and recreation facilities, natural amenities, and percentage of land covered by water were included to represent environmental characteristics of communities at a county level.

### 2.3. Analysis Procedures and Description

Global and local Moran’s I tests were performed to detect the spatial clustered patterns of poor mental health days. The purpose of global Moran’s I analysis was to test the level of spatial autocorrelation in the whole study area associated with poor mental health days. The Moran’s I value ranged from −1 (perfect dispersion) to 1 (perfect clustering) and a zero value indicated a perfect randomness [49,50]. Additionally, local Moran’s I was completed to visualize whether poor mental health days were spatially dependent across the 3109 counties. Among a variety of relevant variables to mental health, “explanatory regression” was adopted to find the models with high explanatory power at the first stage of analysis. Fifteen variables were tested with 45 possible models to find the best linear prediction. Each model also tested multicollinearity, based on a VIF value of 7.5 in ArcMap 10.5 (ESRI, CA, United State). Variables selected for the spatial analysis are listed in Table 1.

A GLM (generalized linear model) was used in the next stage of analysis to test the effects of eight independent variables associated with mental health at a national level. In the final stage of analysis, GWR (geographically weighted regression) analysis was used to explore spatial heterogeneity in the relationships among variables. The GWR supposed that associations between variables may have varied from place to place and created a respective regression coefficient for each analytical unit (e.g., county). The advantage of using “GWR is that an analysis of the spatial variation in model performance and regression coefficients” could enhance both “model specification and understanding of the spatial processes” in the whole and sub areas [51]. Brunsdon et al. [52] and Fotheringham et al. [53,54] suggest a GWR technique because the “parameters are estimated by a weighted least squares procedure. GWR allows local rather than global parameters to be estimated”.

GWR is built on traditional linear regression methods and allows the associations between variables to vary spatially. GWR extended the traditional GLMs by allowing local coefficients to be estimated as follows:

*y_i_ = β_i0_ + β_i1_x_1i_ + β_i92_x_2i_ +……+ β_in_x_ni_ + ε_i_* (1)

The GWR formulation in this study can be rewritten as follows:

(2) $$\begin{array}{cc} \%Poor\;Mental & Health\;Days_{\;i}\\ & =\beta_{0i}\;+\;\underset{1}{\sum }\beta_{1i}Park\;accesibility_{i}+\;\underset{2}{\sum }\beta_{2i}Unemployment_{i}\\ & +\;\underset{3}{\sum }\beta_{3i}\%\;of\;Rural\;area_{i}+\;\underset{4}{\sum }\beta_{4i}Diversity\;index_{i}\\ & +\;\underset{5}{\sum }\beta_{5i}Poverty\;rate_{i}+\;\underset{6}{\sum }\beta_{6i}Education\;attainment_{i}\\ & +\underset{7}{\sum }\beta_{7i}\;{\%\;\mathrm{of}\;\mathrm{Recreation}\;\mathrm{and}\;\mathrm{Fitness}\;\mathrm{Facilities}}_{i}+e_{i} \end{array}$$

The GWR uncovered spatial disparities that were concealed by a single estimation such as used in the GLM (General Linear Model). *β_i0_* was the intercept, and *β_n_* calculated the relationship between the independent variables and the set of i location’s mentally poor days. *ε_i_* was the error related to location i. Locally calibrated coefficients (*β_i_*) varied depending on location (*_i_*), instead of using one single regression coefficient for the variables. The appropriate bandwidth size was selected using the adaptive kernel function. The minimization of the Akaike information criterion determined an appropriate adaptive kernel width for the analysis.

## 3. Results

### 3.1. Spatial Clustered Patterns of Poor Mental Health Days

**Figure 1:**
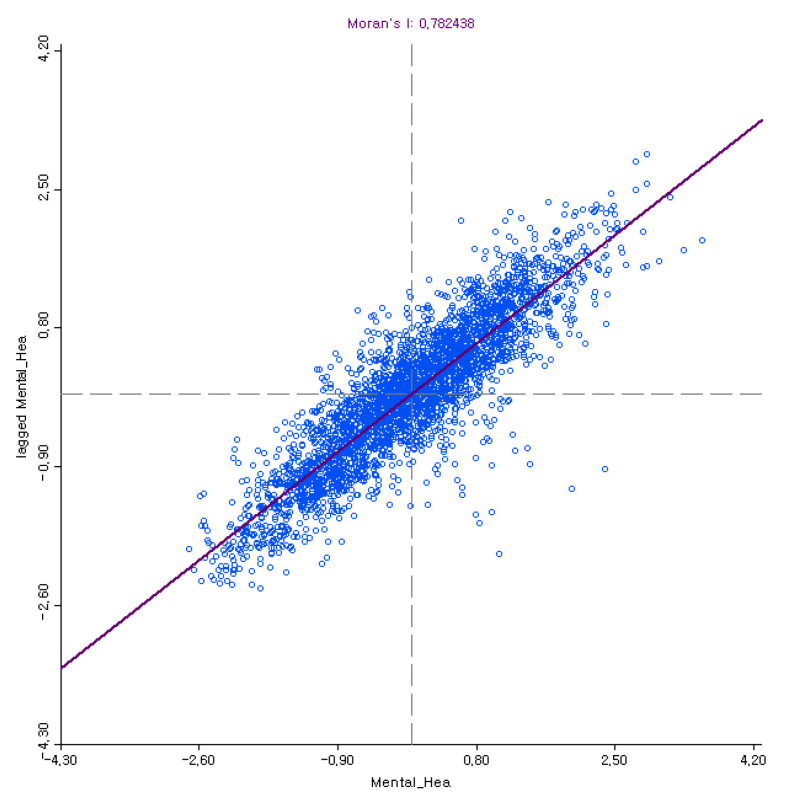
Results of global Moran’s I test.

**Figure 2:**
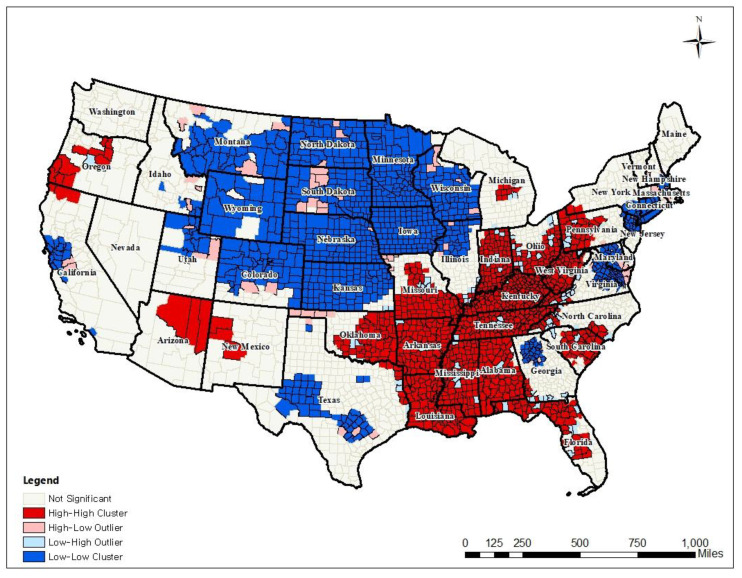
Spatial clustered patterns of poor mental health days.

The global Moran’s I value for mentally unhealthy days was 0.7824 and showed positive autocorrelation in the whole study area (see Figure 1). The local Moran’s I statistics tested and visualized the local spatial cluster patterns of mental health along with a choropleth map (see Figure 2). This showed the relationship between a variable and the corresponding average value in neighboring counties. The visualization of the local Moran’s I results detailed where specific cluster patterns appeared.

Six hundred sixty-seven counties showed high-high (hotspot) clustered patterns in the southeastern part of the U.S. Counties in Alabama, Mississippi, Ohio, Kentucky, and Louisiana showed statistically significant higher levels of poor mental health days than the average values across the study area (*p*-value < 0.001). In contrast, 666 counties in North Dakota, Minnesota, Nebraska, and Wisconsin showed lower levels of poor mental health days as a clustered spatial pattern. This means that strong spatial agglomeration and spatial clustered patterns were seen for poor mental health days.

### 3.2. Generalized Linear Model

The R-squared value ($R^{2}$ = 0.516) of the OLS (ordinary least squares) equation was used for explaining the relationship between poor mental health days and the associated factors with a single equation for the whole study area.

The strongest negative independent variable in the model is park walkability (coefficient −0.376). In addition, density of recreation and sports facilities, college education, diversity index, and the percentage of rural area showed negative association with poor mental health days. Positive associations were found with unemployment and poverty rate (see Table 2 and Table 3).

### 3.3. Spatial Variation of Relationships Between Mental Health and Associated Factors

A geographically weighted regression model was used to figure out spatial variation in the relationships between poor mental health days and associated independent variables determined by stepwise function.

The strength and signals of the associations were specified by the local coefficients. Regression coefficients were not limited to a single variable and were thus able to vary by county in the local regression model. The variations in coefficients uncovered spatial patterns that otherwise would have been hidden. Therefore, local coefficients highlighted the non-stationarity of different factors across the whole study area (see Table 3).

**Figure 3:**
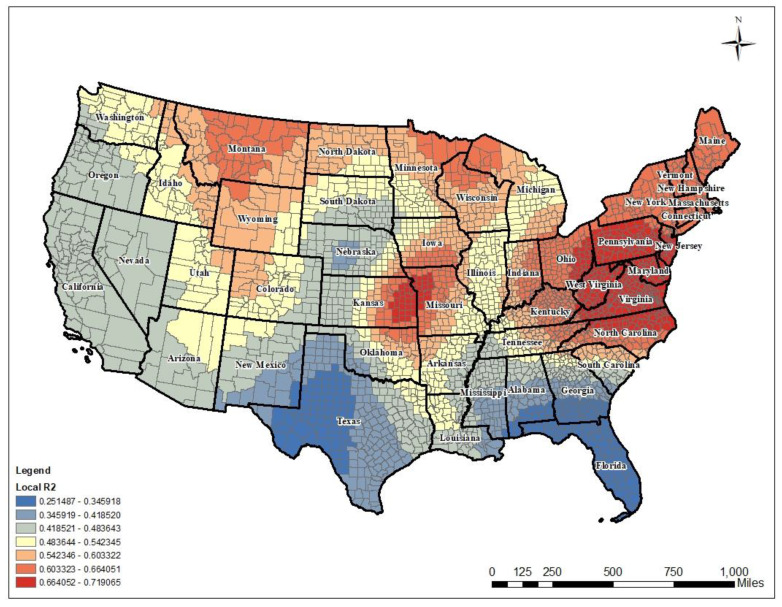
Local R-squared values.

The local $R^{2}$ differed over the U.S. from 25.1% to 71.9% explanatory power. Red-colored areas in Montana, Kansas, and Missouri, and eastern regions such as Pennsylvania, Maryland, Kentucky, Virginia, West Virginia, and Ohio showed that the model better explained poor mental health days via spatial prevalence as compared to GLM in those regions. The GWR accounted for more than 54.2% of the variability. Visualization of local R-squared confirmed that there was non-stationary association across the whole study area (see Figure 3).

Seven independent variables used in GLM showed varied strengths and signals of the relationship in each county.

**Figure 4:**
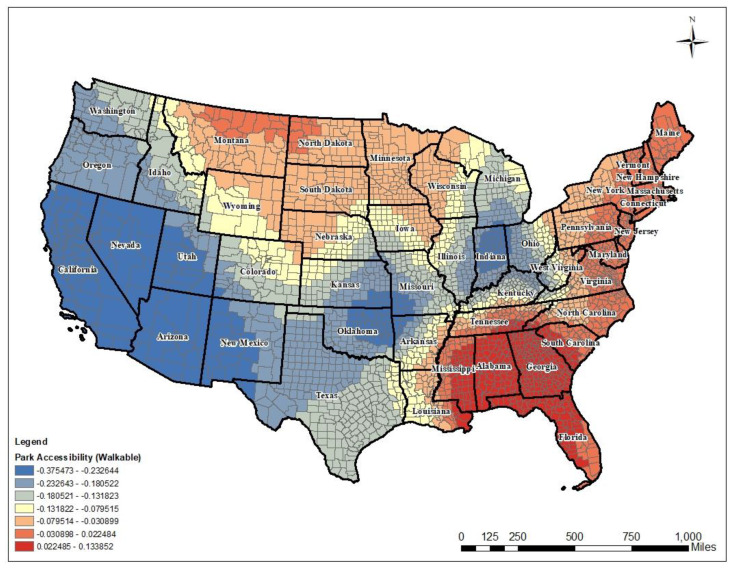
Local coefficients of park walkability.

First, park walkability showed the strongest negative association. The estimated value for the GLM model was −0.376. As residents living within 0.5 miles of parks increased, the average number of poor mental health days decreased. The direction and strength of local coefficients indicated that the influence of park walkability varied significantly across states, with a strong negative influence in the southwestern part of the country, but a strong positive impact in southeastern and northeastern regions. The local coefficient for park walkability ranged from −0.375 to 0.134. Among people who have walkable access to the local parks, residents in the southwestern U.S. were more likely to report fewer poor mental health days than other U.S. regions. Interestingly, southeastern and northeastern U.S. regions showed positive associations with poor mental health days even though residents had walkable access to the local parks (see Figure 4).

**Figure 5:**
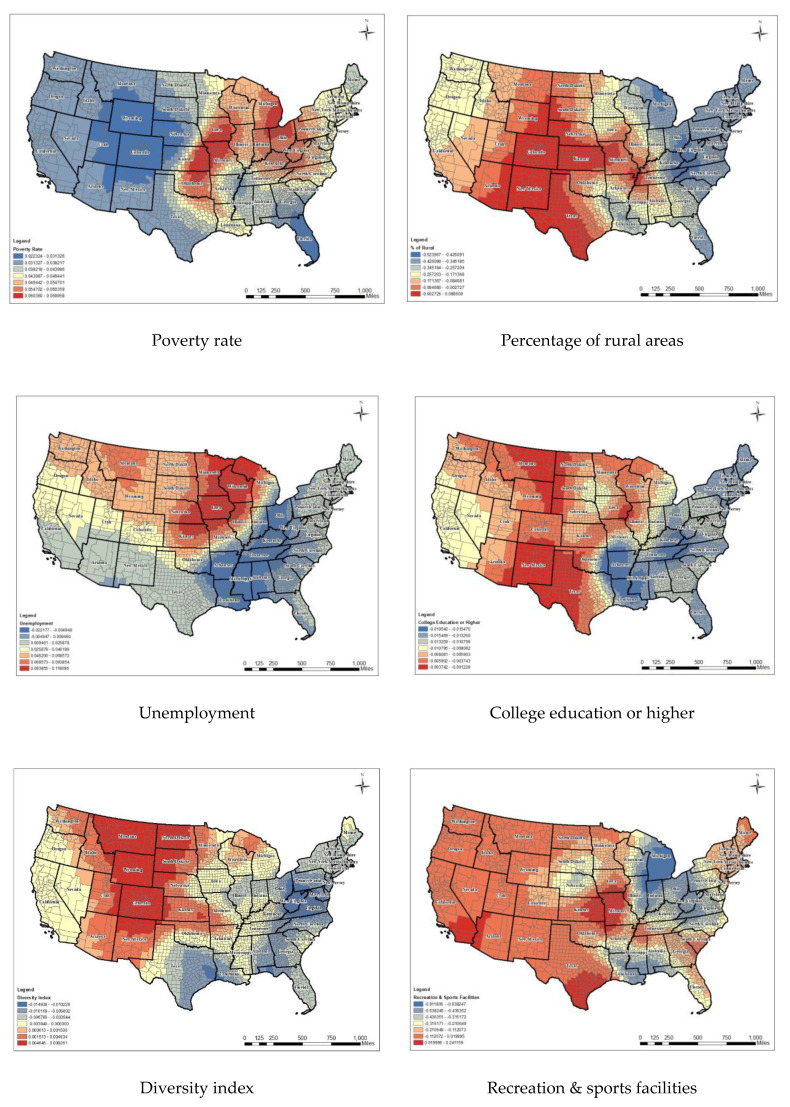
Local coefficients of competing variables in GWR.

Second, there was a positive relationship between poverty rate and poor mental health in GLM (global coefficient: 0.046). In Figure 5, the visualization of local coefficients indicated the influence of poverty rate varied significantly across states, with a strong prevalence in Louisiana, Iowa, Kansas, Oklahoma, Missouri, Ohio, and Michigan (local coefficients from 0.022 to 0.069).

Third, the percentage of rural areas showed a negative association in GLM (−0.337). Figure 5 indicates that local coefficients ranged from −0.524 to 0.089, and both positive and negative associations were found as indicated by the GWR. Counties located in Washington, West Virginia, Kentucky, and Michigan showed strong negative associations, whereas positive associations were found in New Mexico, Colorado, and Kansas.

Fourth, the coefficient of unemployment was 0.056 in GLM. Local coefficients ranged from −0.022 to 0.118. Counties located in Ohio, Kentucky, Tennessee, Mississippi, and Arkansas showed strong negative associations, whereas positive associations were found in Iowa, Wisconsin, and Michigan.

Fifth, college education (B.A. degree or higher) was negatively associated with poor mental health days in GLM (global coefficient: −0.009). Overall, the weakest relationship in the model was educational attainment. The local coefficient ranged from −0.019 in Louisiana, Arkansas, Kentucky, Tennessee, and Mississippi to −0.001 in New Mexico, the western part of Texas, and the eastern part of Montana and Iowa.

Historically, unemployment levels and poverty rates have been regarded as contributing factors for serious symptoms of depression requiring medical treatment. In addition, links between higher educational attainment and lower risk for depression have been confirmed by previous studies [55,56].

Sixth, the diversity index ranged from 0 (no diversity) to 100 (complete diversity). If the entire population in the community belongs to “one racial/ethnic group, then an area has zero diversity; the diversity index increases to 100 when the population is evenly divided into two or more racial/ethnic groups” [57]. This variable could represent residential segregation and its association with mental health conditions. In GLM, the diversity index showed negative association with poor mental health days (coefficient: −0.004), suggesting that dynamic components of racial/ethnic groups may positively impact residents’ mental health. In addition, GWR detected different spatial patterns of coefficient for the diversity index between the west side and east side of a country. Positive associations were detected in western regions such as Montana, Colorado, Wyoming, and western parts of North Dakota, whereas negative associations appeared in eastern parts of Texas and the east coast of the U.S. simultaneously.

Seventh, the percentage of recreation and sports facilities was negatively associated with poor mental health days in GLM (global coefficient: −0.026). A mix of positive and negative relationships was found for the percentage of recreation and sports facilities in the GWR model. Counties located in Ohio, Kentucky, Tennessee, Mississippi, and Arkansas showed strong negative associations, whereas positive associations were found in Missouri and the southern parts of Texas and California.

## 4. Discussions

The built and social environments have many features that result from, or are part of, how humans interact with each other [58,59,60]. These include socioeconomic elements such as poverty rate, income level, and other similar factors [61,62]. The surrounding environment can correlate with income and poverty, which can affect an individual’s ability to live adjacent to parks and other environmental amenities [63,64].

Large-scale regional parks might be visited by people from long distances away, but most neighborhood parks are primarily used by local residents. In the same way, surveys of residents insist that most would not use a park unless it was within a quarter-mile of their residential area. Previous studies suggested that people who live in denser communities and those who have nonresidential land uses within walking distance of their homes are more likely to walk. Similarly, most users of neighborhood parks are people who live within walking distance of these amenities and are people would not walk without a place to walk to. This highlights the importance of harmonizing land use and transportation [65].

The findings in this study revealed that park proximity (within walking distance) was among the most important factors for decreasing poor mental health days. A local park is an important component in the regional recreation system and plays a key role in providing balanced recreation service provision and opportunities. The strengths of this study are that it represents a comprehensive examination of the continental United States with GML and provides spatially varying relationships across sub-regions with GWR. In social science fields, the use of GML has been popular, based on solid theoretical justification (theory-driven), to examine relationships. However, it should not be overlooked that different relationships based on different geographical settings could exist beyond the single-theory explanation. To cover this previous limitation, GWR detected locally calibrated regression coefficients to suggest new associations with poor mental health days at a county level. Interpretation of the results was more complex compared to GML. However, practitioners need more specific information to make effective decisions in complex real-world situations.

To best implement the study findings, it is important to stress that well-maintained parks could be assets for the neighborhoods around them. They may increase the livability of a community and contribute to residential stability and increased property values. Parks have provided an opportunity for community members to interact. Some parks may have issues with crime, drug use, or other problematic behaviors; if neighborhoods perceive a park as unsafe, they may not use it. Park maintenance is critical if parks are to be perceived and used as community assets. These facilities should be designed to have age-appropriate equipment, accommodate different groups, and have equipment that meets current safety standards. These recreational spaces must be well maintained, and if necessary, should be sensitive to issues of crime and safety. Mixed signals in GWR models for park walkability may stem from maintenance conditions of neighborhood parks. The other possible reason for variation of local coefficients could be “concentrated poverty”, which are areas that have high rates of poverty and a low diversity index (residential segregation based on single racial/ethnic group). It also refers to the potential problem that people in these neighborhoods have little contact with those who are not poor. In our findings, poverty rate showed positive associations with poor mental health days at varying strengths of relationship. Future researchers should further explore racial/ethnic residential segregation and test the association with mental health conditions in the community.

It is important to address the fact that the current study used a 2017 cross-sectional data module of the BRFSS. Thus, the findings in this study could not determine causality, but could explain associations in the relationship between walkable recreation opportunities and poor mental health days while controlling for a variety of competing explanations (e.g., socioeconomic status). In addition, the interpretation of the findings is limited because this study did not differentiate characteristics of neighborhood parks (e.g., the types of park, maintained condition of park, or size of park) nor did it include longitudinal follow-up. Future studies should consider a longitudinal path to explore the change in leisure-related factors and community-level mental health. This data consisted of county-level information, the smallest analytical unit currently available at a national level across multiple years. Due to the nature of the secondary data and local multiclonality problems, the selection of measures was beyond the researchers’ control. Future research might further examine the place-based associations with mental health using census-tract data within these regions or by applying methods such as longitudinal and qualitative research at the local level. Finally, neighborhood parks are integral in providing opportunities for physical activity and may also play a role in improving the mental health condition of people [66]. However, the direct relationship between park walkability and participation in physical activities was not examined in this study. Future studies are needed to better understand the influence of park walkability on physical activities and the connection between recreation opportunities and public mental health.

## 5. Conclusions

To date, there are no rigorous studies on the relationships between population-based mental health condition and recreation opportunities at a national level. This study is the first study to undertake a mixed spatial modeling for exploring park walkability and its association with mentally unhealthy days. This study examined if park walkability has an effect on the number of mentally unhealthy days after controlling for a variety of competing explanations. The findings suggest that multiple factors associated with poor mental health days, particularly walkable access to local parks, showed strong explanatory power in both GLM and GWR models. In addition, negative relationships were found with educational attainment, racial/ethnic dynamics, and lower levels of urbanization. Positive relationships were found with poverty rate and unemployment in the GLM. Finally, GWR detected variance in the strength and direction of associations for 3109 counties. Thus, using both GLM and GWR analysis (national and county level) provides a comprehensive basis for informing effective recreation planning across spatial and regional scales. These results may address the gaps in those previous studies focused on individual-level scope and those without a spatial context.

## Figures and Tables

**Table 1:** Variables and data sources used for the final model.

Name of Variable	Description	Source
**Dependent Variable**		
Poor mental health days	County level age-adjusted prevalence of poor mental health days (average days in past 30 days)	Behavioral Risk Factor Surveillance System, 2017
**Independent Variables**		
% of Rural area	Percentage of rural areas	Census Population Estimates, 2010
Poverty rate	The ratio of people whose income falls below the poverty line	United States Department of Agriculture (ERS), 2014
Unemployment	Percentage of unemployment	Bureau of Labor Statistics, 2016
Educational attainment	Percentage of individuals who obtained a college degree or higher	American Community Survey, 2015
Diversity index	Level of racial and ethnic diversity	ESRI Demographics, 2016
Park walkability	Residential proximity to parks within 0.5 mile	National Environmental Public Health Tracking Network, 2015
% of Recreation and fitness facilities	Recreation & fitness facility/1000 population	U.S. Census Bureau’s County Business Pattern (CBP), 2012

**Table 2:** Results of the generalized linear model (GLM).

Variable	Coefficient	StdErr	t Statistic	RobustSE	Robust_t,	Robust_Pr	VIF
Intercept	3.575	0.054	66.547	0.0557	64.165	0.000 *	
Unemployment	0.056	0.003	19.037	0.0037	14.863	0.000 *	1.389
B.A or higher degree	−0.009	0.001	−7.979	0.001	−8.623	0.000 *	1.827
% of Recreation & sports facilities	−0.266	0.082	−3.256	0.0823	−3.235	0.001 *	1.111
Diversity index	−0.003	0.0004	−8.547	0.0004	−8.275	0.000 *	1.407
Poverty rate	0.046	0.002	28.340	0.002	351.538	0.000 *	1.719
Park walkability	−0.376	0.044	−8.573	0.043	−8.744	0.000 *	1.206
% of Rural areas	−0.337	0.032	−10.656	0.0322	−10.446	0.000 *	1.763

Koenker (BP) Statistic: 181.231134 Prob (>chi-squared), (6) degrees of freedom: 0.000000 *; Jarque-Bera Statistic: 26.722466 Prob (>chi-squared), (2) degrees of freedom: 0.000002 *.

**Table 3:** Comparison results between ordinary least squares (OLS) and geographically weighted regression (GWR).

	OLS	GWR			
		Mean	Minimum	Maximum	Standard Deviation
Intercept	3.575 *	32.539	27.066	38.932	2.502
Unemployment	0.056 *	0.030	−0.022	0.118	0.0396
Educational attainment	−0.009 *	−0.009	−0.019	−0.001	0.004
Diversity index	−0.004 *	−0.004	−0.013	0.008	0.006
Poverty rate	0.046 *	0.046	0.022	0.069	0.010
Park walkability	−0.376 *	−0.099	−0.375	0.134	0.096
% of Rural areas	−0.337 *	−0.205	−0.524	0.089	0.162
% of Recreation & sports facilities	−0.266 *	−0.218	−0.912	0.241	0.197
Local $R^{2}$		0.513	0.285	0.702	0.094
R^2^	0.516	0.764180			
Adjusted $R^{2}$	0.515	0.755769			
AIC (Akaike Information Criterion)	3396.467	1309.86037			
Koenker statistics	181.231 *	Neighbors: 797			
Jarque-Bera statistics	26.722 *	Bandwidth methods: AICc			
		Kennel type: Adaptive			

* *p* < 0.001.
